# The Puerperal Diseases

**Published:** 1874-05

**Authors:** 


*The Puerperal Diseases; Clinical Lectures delivered at Bellevue Hospital.* By Fordyce Barker, M. D., Clinical Professor of Midwifery and the Diseases of Women, in Bellevue Hospital Medical College, etc. New York: D. Appleton & Co., 1874. Buffalo: Martin Taylor.

We have not had the pleasure in some time of reading a book which has seemed to supply a want in the profession so much as the one under consideration. The increased importance which is being daily attached to the obstetric department, has created a demand for a book of this character, which the author has attempted to fulfill in the form of a series of clinical lectures upon the diseases incident to the puerperal state.

The reader is conveyed in these lectures to the bedside of the patient, and seems to see the case under consideration before him. The book is composed of twenty lectures, which treat of Puerperal Convalesence, Diet of Puerperal, Women, Lacerations of the Perineum, Thrombus of the Vulva and Vaginal Puerperal Albuminuria, Convulsions, Lactation, Mastitis and Mammary Abscess, Puerperal Mania, Relaxation of the Pelvic Symphysis, Phlegmasia Dolens, Puerperal Phlebitis, Puerperal Metritis, Puerperal Peritonitis, Pelvic Peritonitis and Cellulitis, Septiæmia and Pyæmia, Puerperal Fever.

This book will be read and re-read by all who are its fortunate possessors, and we do not hesitate to say that all will agree with us when we say that it is one of the most valuable works which has been written by an American author in some time. There are points upon which those who read this work will find it hard to agree with the author ; they are few, however, and in every instance the author has called to his aid the teachings of a long and rich experience before expressing his opinions. We commend the work to the careful attention of all medical practitioners as one which is eminently worthy of their thoughtful study and consideration.

